# HIF1α-dependent induction of the mitochondrial chaperone TRAP1 regulates bioenergetic adaptations to hypoxia

**DOI:** 10.1038/s41419-021-03716-6

**Published:** 2021-05-01

**Authors:** Claudio Laquatra, Carlos Sanchez-Martin, Alberto Dinarello, Giuseppe Cannino, Giovanni Minervini, Elisabetta Moroni, Marco Schiavone, Silvio Tosatto, Francesco Argenton, Giorgio Colombo, Paolo Bernardi, Ionica Masgras, Andrea Rasola

**Affiliations:** 1 Dipartimento di Scienze Biomediche, Università di Padova, viale G. Colombo 3, 35131 Padova, Italy; 2 Dipartimento di Biologia, Università di Padova, viale G. Colombo 3, 35131 Padova, Italy; 3 Istituto di Scienze e Tecnologie Chimiche, CNR, Via Mario Bianco 9, 20131 Milano, Italy; 4 Dipartimento di Chimica, Università di Pavia, via Taramelli 12, 27100 Pavia, Italy; 5 Istituto di Neuroscienze, CNR, Viale G. Colombo 3, 35131 Padova, Italy

**Keywords:** Cancer metabolism, Cell signalling

## Abstract

The mitochondrial paralog of the Hsp90 chaperone family TRAP1 is often induced in tumors, but the mechanisms controlling its expression, as well as its physiological functions remain poorly understood. Here, we find that TRAP1 is highly expressed in the early stages of Zebrafish development, and its ablation delays embryogenesis while increasing mitochondrial respiration of fish larvae. TRAP1 expression is enhanced by hypoxic conditions both in developing embryos and in cancer models of Zebrafish and mammals. The TRAP1 promoter contains evolutionary conserved hypoxic responsive elements, and HIF1α stabilization increases TRAP1 levels. TRAP1 inhibition by selective compounds or by genetic knock-out maintains a high level of respiration in Zebrafish embryos after exposure to hypoxia. Our data identify TRAP1 as a primary regulator of mitochondrial bioenergetics in highly proliferating cells following reduction in oxygen tension and HIF1α stabilization.

## Introduction

Changes in metabolic circuities are required by highly proliferating cells during development or tumorigenesis to fuel biosynthetic pathways^1,2^, and central carbon metabolism coordinates a variety of reactions to ensure the generation of energetic molecules and anabolic building blocks even in conditions of scarce oxygen availability^3,4^. To cope with hypoxia, tumor cells can downregulate oxidative phosphorylation (OXPHOS) while increasing their metabolic flux through glycolysis and pentose phosphate pathway (PPP), in a bioenergetic rewiring recalled as aerobic glycolysis or Warburg effect^5^ that allows cells to maintain a high proliferative capacity by providing nucleotides, amino acids and NADPH for anti-oxidant defenses^6^. Similar metabolic adaptations are also enacted to boost proliferation during early developmental stages in organisms as diverse as Zebrafish (*Danio rerio*), fruit fly (*Drosophila melanogaster*), African clawed frog (*Xenopus laevis*) and mammals^2,7,8^. Only later in embryogenesis, the building of the cardiovascular system increases oxygen tension and leads to a concomitant OXPHOS acceleration^9^.

Under limited oxygen availability, the hypoxia-inducible factor HIF1α is stabilized and orchestrates a complex transcriptional program that further shapes cell metabolism by inducing glycolysis and repressing OXPHOS^9–11^, promotes cell proliferation and motility and also elicits angiogenesis^12^. Therefore, HIF1α activation is crucial in many settings of neoplastic growth and during embryonic development; indeed, its silencing causes embryonic lethality or developmental defects, depending on the animal model^13–15^. We have demonstrated that HIF1α can be stabilized by TRAP1, the mitochondrial paralog of the HSP90 chaperone family, through inhibition of succinate dehydrogenase (SDH) and the subsequent increase in intracellular succinate concentration that abrogates the priming for proteasomal degradation of HIF1α^16,17^. TRAP1 induction of HIF1α is pseudohypoxic, as it occurs independently of oxygen availability, and supports a pro-neoplastic metabolic shift toward aerobic glycolysis in a variety of tumor cell types^16^. Thus, it constitutes an example of how mitochondria can tune the metabolism of proliferating cells, allowing their rapid adaptations to fluctuating environmental conditions. However, it remains poorly investigated the role played by TRAP1 in determining the metabolic changes of cells under physiological conditions, as well as the molecular mechanisms regulating its expression. In this respect, embryonic development, in which cells undergo a fast proliferation rate under varying conditions of oxygen tension^2^, represents an interesting field of investigation for TRAP1-dependent regulation of cell bioenergetics.

Zebrafish (*Danio rerio*) is a good model to investigate metabolic features of a developing organism as many of its biochemical pathways are conserved with mammals and its embryonic developmental stages have been extensively characterized^18,19^. Mitochondrial OXPHOS progressively increases during Zebrafish development through poorly characterized mechanisms^20^. Here, we report that this metabolic change is accompanied by a parallel decrease in HIF1α and TRAP1 levels. We find that TRAP1 is induced by HIF1α and acts as a major repressor of OXPHOS under hypoxic conditions. These observations apply both to embryonic development and to fish and mammal tumor models.

## Materials and methods

### Animals

Zebrafish embryos were maintained in a temperature-controlled room (28.5 °C) and fed as described by Kimmel et al.^21^. Fish were kept under a 14 h light-10 h dark cycle. For mating, males and females were separated in the late afternoon and the next morning were freed to start courtship, which ended with egg deposition and fecundation. Eggs were collected, washed with fish water (0.5 mM NaH_2_PO_4_, 0.5 mM NaHPO_4_, 0.2 mg/l methylene blue, 3 mg/l instant ocean) and maintained at 28.5 °C in fish water supplemented with an antibiotic-antimycotic cocktail (50 μg/ml ampicillin, 100 units/ml penicillin, 0.1 mg/ml streptomycin and 3.3 μg/ml amphotericin B). The male Zebrafish reporter line for HIF1α Tg(4xhre-tata:eGFP)^ia^^21,22^ was outcrossed with female wild-type Zebrafish, and green heart positive offspring embryos were selected and used for experimental purposes. To generate a Zebrafish model of pancreatic adenocarcinoma, one-cell stage eggs derived from Tg(ptf1a:Gal4) outcrossed with wild-type Zebrafish were injected with Tol2(UAS:eGFP-KRAS^G12D^) plasmid, generating the final transgenic line Tg(ptf1a:Gal4)/UAS:eGFPKRAS^G12D^, as described^23^. Approximately 100 embryos were raised, all expressing eGFP according to the expected ptf1a pattern. eGFP expression was evaluated from 1-week intervals to 1 month. Positive fish expressing eGFPKRAS^G12D^ were then monitored until the development of a tumor mass, when fish were euthanized for further histological and biochemical analyses. Fish were photographed live using a NIKON C2 H600L confocal microscope with ×20 and ×40 water dipping objectives. Laser used to excite fluorophores was 488 nm for eGFP. To generate Zebrafish TRAP1 knock-out model, eggs were injected at one-cell stage and analysed at 24, 48 hours post-fertilization (hpf) and at 1-month post fertilization. The developmental stages at which experiments were carried out are indicated in the main figures and are within the fifth day post-fertilization (dpf).

### Human cell lines

Experiments were performed on human U87 and U251 glioblastoma cells, human pancreatic carcinoma cells (MiaPaCa-2 and PANC1), human pancreatic adenocarcinoma cells (BxPc3), and human ipNF95.6 plexiform neurofibroma cells (kindly provided by Dr. Margaret R. Wallace, University of Florida, College of Medicine, Gainesville, FL). U87 and U251 cells were grown in minimum essential medium (MEM) supplemented with 10% fetal bovine serum (FBS), 1% glutamine, 1% sodium pyruvate, 1% non-essential amino acids, and 1% penicillin and streptomycin. MiaPaCa-2, PANC1, BxPc3, and ipNF95.6 cells were grown respectively in Dulbecco’s Modified Eagle’s Medium (DMEM) and RPMI-1640 supplemented with 10% FBS 100 units/ml and 1% penicillin and streptomycin. All cells were cultured at 37 °C in a humidified atmosphere containing 5% CO_2_.

### sgRNA template design and generation

The CRISPR/Cas9 mediated mutagenesis of TRAP1 gene was achieved by applying the protocol proposed by Gagnon et al.^24^. CHOPCHOP (https://chopchop.cbu.uib.no/) was used to identify the sgRNA sequence against the exon 6 of TRAP1 gene. For gene-specific oligonucleotide design, the SP6 promoter sequence was added before the sgRNA followed by an overlap sequence, which interacts with the 80 bp long constant oligo (Supplementary Table 1). The dsDNA was generated by using the T4 DNA polymerase (NEB M0203S) after annealing of gene-specific oligonucleotide and the constant oligo. The dsDNA template was then purified by using the PCR clean up column (Quiagen 28704) and transcribed over night at 37 °C with the Ambion Megascript SP6 KIT (Ambion AM1330). The resulting sgRNA was purified with RNA clean and concentrator Kit (Zymo Research R1017), quantified, and stored at −80 °C.

### sgRNA and Cas9 injection and determination of somatic mutagenesis rate

Zebrafish (Tübingen) eggs were collected and injected with a mix composed of sgRNA (70 ng), Cas9 protein (NEB M0641S), and phenol red injection dye. The sgRNA and Cas9 were incubated for 5 min at room temperature to form the complex. Embryos were injected soon after the fertilization and kept at 28.5 °C. The day after injection, the genomic DNA was extracted from 24 hpf injected embryos. Single fish were incubated 20 min with NaOH 50 mM at 95 °C, then cooled on ice followed by the addition of Tris-HCl 1 M, pH 7.5. The extracted DNA was amplified by PCR using Phusion High-fidelity Master mix (M0531S) and relative primers. We then performed heteroduplex mobility assay (HMA) to identify the presence of somatic mutations. Briefly, the PCR products were mixed with HMA buffer (1 M NaCl, 100 mM Tris (pH 7.8), and 20 mM EDTA) and gel loading buffer 1X. Samples were denatured at 95 °C for 15 min, then placed 15 min at room temperature and cooled on ice. Samples were loaded in a 10% polyacrilammide gel in TBE (boric acid 89 mM; EDTA 2 mM, pH 8.3) at 30 mA for 2 h. Homoduplexes and heteroduplexes were visualized following staining of the gel with SYBR Green II nucleic acid stain (Invitrogen).

### Determination of germline mutations and generation of adult fish homozygotes

Upon sgRNA activity evaluation in somatic tissue, the remaining embryos were grown in order to identify germline-transmitting founders (F_0_). Each founder was outcrossed with wild-type Tübigen Zebrafish, and then 15 embryos from each pair were screened for mutations in F_1_. DNA was extracted and amplified by PCR. Possible mutations were detected with HME on PCR products as described above. Although HMA is a rapid method to screen mutants, it does not provide the exact nature of the mutation. The screened alleles must be sequenced in order to get precise information on the mutation. Moreover, sequencing allows discarding those mutations that do not result in the introduction of a stop codon cassette. The positive F_1_ fish were raised to adulthood and crossed again with wild-type ones to generate the F_2_ population. Heterozygous were identified by PCR and sequencing of DNA extracted from tail at 1 month post fertilization. This scheme was used to generate F_3_ heterozygotes fish. Then, the F_3_ heterozygotes fish were inbred, and the homozygotes were identified by DNA extraction from tail at 1 mpf followed by PCR and sequencing analysis

### Generation of HIF1α knock-down cell lines

HIF1α knock-down cells were generated by using the short hairpin RNA strategy in U87 cell line. Sequences for the HIF1α shRNA (shRNA10819 5′-CCGGTGCTCTTTGTGGTTGGATCTACTCGAGTAGATCCAACCACAAAGAGCATTTTT-3′ and shRNA3809 5′- CCGGCCAGTTATGATTGTGAAGTTACTCGAGTAACTTCACAATCATAACTGGTTTTT-3′) were obtained from Sigma Aldrich. Scrambled shRNAs were used as negative controls. shRNA oligonucleotides were co-transfected with the packaging plasmids psPAX (Addgene #12260) and pMD2.G (Addgene, #12259) into the human embryonic kidney (HEK) 293T cells for viral production. Recombinant virus was collected and used to infect cells by standard methods. Infected cells were then selected with 2 µg/ml puromycin.

### Drug administration to fish and exposure to hypoxic conditions

Zebrafish embryos were maintained in hypoxia (5% O_2_) for the indicated time in a temperature-controlled room (28.5 °C). Dimethyloxalylglycine (DMOG, 100 μM or 150 μM), dimethyl succinate (DMS, 1 mM) and TRAP1 inhibitor compound 5 (100 μM) were dissolved in fish water and maintained for the indicated time at 28.5 °C. Succinate (0.5 μM and 1 mM) was dissolved in fish water every day from 24 hpf until the experimental time point. U87, U251, MiaPaCa-2, BxPc3, and ipNF95.6 cells were incubated in a hypoxic chamber (Baker Workstation InVIVO_2_ I400) at 0.5% O_2_, or treated with 0.5 mM CoCl_2_ or 100 μM TRAP1 inhibitor compound 5 for the indicated times.

### Muscle birefringence analysis

Muscle birefringence was analyzed in zebrafish embryos at the indicated time point. Briefly, embryos were anesthetized with 0.02% tricaine Tris-Cl pH 9.1, embedded in 2% methylcellulose, and placed on the glass. Muscle light refraction was analyzed using a Leica S9i stereomicroscope, equipped with a camera and two polarizing filters, the same exposure settings and magnification was used for both wild-type and TRAP1 knock-out fish. Muscular birefringence was calculated by dividing the mean intensity by the selected area of the fish using ImageJ software as described^25^.

### Epiboly, fish area, pigment measurements, and cardiac frequency

To estimate epiboly and fish area the cell layer or the total fish body, respectively, were analyzed by excluding the yolk. For pigment analysis the trunk region was considered and the number of pigments was counted by using ImageJ software. All pictures were taken with Leica S9i microscope and analyzed with ImageJ software. Fish area, epiboly, and number of pigments are average ±SEM of the indicated embryos per condition.

For cardiac frequency analysis, Zebrafish larvae were embedded in 3% methylcellulose without using anesthetic to avoid any tricaine-dependent effect on the cardiac rate. Beats per minute (BPM) were calculated by averaging the beats counted four times in 15 s. Cardiac frequency is reported as average ±SEM of 10 animals per condition.

### Fish imaging

For bright field imaging, fixed larvae were embedded in 1% methylcellulose and images were acquired with a Leica M165 FC microscope equipped with a Nikon DS-Fi2 digital camera. Fluorescence imaging was performed embedding fixed larvae with 1% low-melting agarose and mounted on a depression slide; Nikon C2 confocal system was used to acquire images.

### In situ RNA hybridizations

Whole-mount RNA in situ hybridization on zebrafish embryos was performed as described^26^. WT and TRAP1 knock-out larvae were hybridized in the same tube in order to avoid method-dependent artifacts. Trypsin and prox1 probes were obtained as previously described^27,28^.

### Behavioral assays

For behavioral assays, 72 hpf, 96 hpf, and 120 hpf Zebrafish were placed in 24-well plates with 1 ml of fish water and images recorded with DanioVision tracking system (Noldus Information Technology, Wageningen, The Netherlands). After 20 min of acclimation, larvae movement was recorded repeating three cycles of 10 min of light and 10 min of dark^29^. Locomotor activity was analyzed using Ethovision 11 software (Noldus Information Technology, Wageningen, The Netherlands).

### Protein isolation and western blot analysis

For Western immunoblot analyses, embryos or cells were lysed at 4 °C in an RIPA buffer composed of Tris-HCl 50 mM pH7.4, NaCl 150 mM, NP40 1%, sodium deoxycholate 0.5%, SDS 0.1%, EDTA 2 mM and protease inhibitors (Sigma). Lysates were then clarified at 14,000 rpm for 30 min at 4 °C and quantified by using a BCA Protein Assay Kit (Thermo-Scientific). Protein extracted from embryos, tissue, or cells were boiled at 50 °C with Laemli buffer for 5 min, separated in reducing conditions by using NuPage Novex 4%-12% Bis-Tris gels (Life Technologies) and transferred in Hybond-C Extra membranes (Amersham). Primary antibodies were incubated for 16 h at 4 °C. Proteins were visualized using the UVITEC imaging system following incubation with horseradish peroxidase-conjugated secondary antibodies.

### RNA extraction and RT-PCR

Total mRNA was isolated from Zebrafish embryos by using TRIZOL (Invitrogen). Two micrograms of total mRNA were retro-transcribed with Superscript (Invitrogen). qPCR of the indicated genes (Supplementary Table 1) was performed by using GoTaq qPCR Master Mix (Promega).

### Measurement of succinate: coenzyme Q reductase (SQR) activity of SDH

To measure SDH activity, embryos were homogenized in an assay buffer composed of KH_2_PO_4_ (25 mM, pH 7.4) and MgCl_2_ (5 mM) containing protease and phosphatase inhibitors (Sigma Aldrich). Total lysates were then quantified using a BCA Protein Assay Kit (Thermo-Scientific). Sixty micrograms of total homogenate were incubated for 10 min at 30 °C in the assay buffer supplemented with sodium succinate 20 mM and alamethicin 5 μM. After incubation, a mix composed of sodium azide 5 mM, Antimycin A 5 μM, Rotenone 2 μM, and Coenzyme Q1 65 μM was added. SDH activity was measured by following the reduction of 2–6 dichloro phenolindophenol (DCPIP) at 600 nm (ε = 19.1 nM^−1^ cm^−1^) at 30 °C. Inhibitors (17AAG or compound 5) were added 5 min before starting recordings.

SDH enzymatic activity was also assessed histochemically on frozen tissue. Soon after extraction, pancreatic tissue was embedded in frozen OCT (Kaltek 0782). Tissue sections were incubated 30 min at 37 °C with an incubation medium containing KH_2_PO_4_ 0.2 M, pH 7.4, sodium succinate 0.2 M, and nitro blue tetrazolium (NBT) salt (N6876 Sigma-Aldrich). The TRAP1 inhibitor compound 5 (100 μM) was included in incubation media were indicated. Sections were then rinsed with PBS 1× and covered by using a mounting medium. Succinate was oxidized to fumarate and reduction of the final electron acceptor NBT allows the formation of purple staining.

### Measurement of oxygen consumption rate (OCR)

OCR was measured with Seahorse XF24 extracellular flux analyzer (Agilent) in living Zebrafish embryos at 96 hpf and in human U87 glioblastoma cell line. Zebrafish embryos were placed into XF24 microplate well (1 embryos per well) and blocked with a capture screen to keep them in place; 670 μl of fish water (0.5 mM NaH_2_PO_4_, 0.5 mM NaHPO_4_, 3 mg/l instant ocean) was added. The basal respiration was measured for 130 or 68 min at 28.5 °C. FCCP (0.5 μM) was used to measure maximal respiration while Rotenone (0.5 μM) and Antimycin A (5 μM) were used to completely abolish mitochondrial respiration. Respiratory rates are average ±SEM of the indicated embryos per condition.

### In silico analysis of TRAP1 promoter

The TRAP1 nucleotide sequences were retrieved from Ensembl^30^ (accession codes: ENSG00000126602 and ENSDARG00000024317 for human and zebrafish, respectively), aligned with BLASTn^31^ and visualized with Jalview^32^. Conserved HREs were extracted through the bash shell command line, while transcription factors binding within the human TRAP1 promoter region were retrieved from GeneCards^33^. HIF1α interactors were extracted from the STRING^34^.

### In vivo measurements of ROS levels

ROS levels were measured by incubating WT and TRAP1 knock-out Zebrafish larvae at 96 hpf in Hank’s Balanced Salt Solution (HBSS) and MitoSOX probe for 30 min at 30 °C; three washes of ten minutes were then performed. Red fluorescence emitted by mitoSOX probe was measured by acquiring live embryo images with a Nikon C2 H600L confocal equipped with ×40 water immersion objective, camera, and laser emitted at 561 nm. ROS signal was analyzed by measuring the integrated density of emitted fluorescence in the brain region normalized for the area analyzed with ImageJ.

### Antibodies

Mouse monoclonal anti-rodent TRAP1 was from Becton Dickinson (Cat. #612344); mouse monoclonal anti-human TRAP1 was from Santa Cruz (Cat. #sc-73604); mouse monoclonal anti-β actin was from Santa Cruz (Cat. #sc-47778); rabbit polyclonal anti-citrate synthetase was from Abcam (Cat. #ab96600); rabbit polyclonal anti-HIF1α was from Novus Biologicals (Cat. #NB100-449); rabbit polyclonal anti-HIF1α was from GeneTex (Cat. #GTX131826); rabbit polyclonal anti-phospho-ERK1/2 was from Cell Signaling (Cat. #9101); rabbit polyclonal anti-glucose transporter GLUT1 was from Abcam (Cat. #ab652).

### Quantification and statistical analysis

Data were analyzed and presented as mean ± standard error of the mean (SEM) in all figures. Pairs of data groups were analyzed using paired and unpaired two-tailed Student’s *t* tests. In the case of more than two groups, a one-way analysis of variance (ANOVA) followed by Bonferroni post hoc test was applied. Statistical significance was determined using GraphPad Prism 8. Results with a *p* value lower than 0.05 compared to controls were considered significant and indicated as ^∗∗∗^*p* < 0.001, ^∗∗^*p* < 0.01, ^∗^*p* < 0.05. Each experiment was repeated at least three times.

## Results

### Absence of TRAP1 affects early stages of Zebrafish embryogenesis

**Fig. 1:**
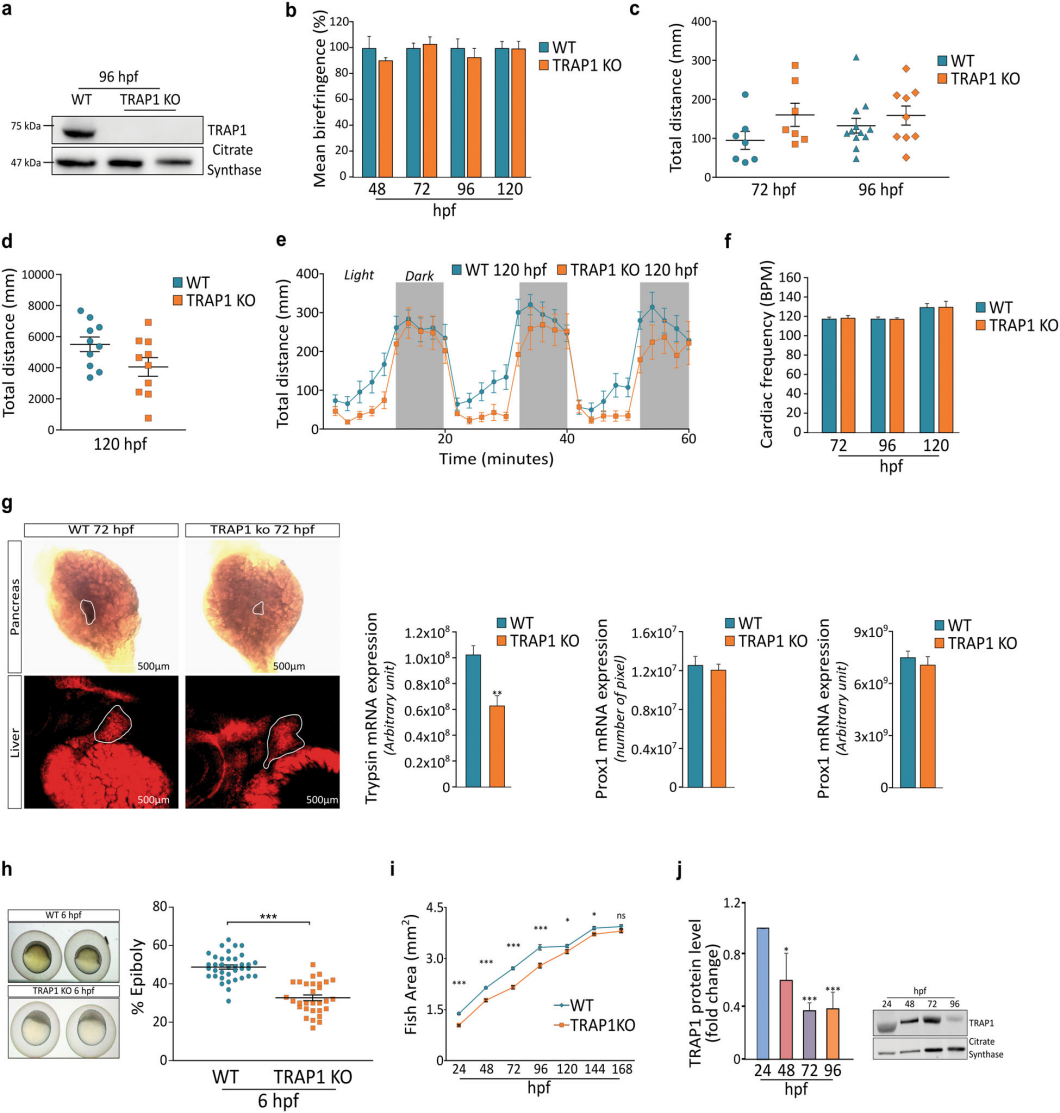
Characterization of Zebrafish TRAP1 Knock-out fish. **a** Western blot analysis of TRAP1 protein level in wild-type and knock-out animals at 96 hpf. The mitochondrial protein citrate synthase was used as a loading control. **b** Quantification of Zebrafish embryos birefringence from 48 hpf to 120 hpf; data are presented as percentage of muscle birefringence normalized for the fish area for at least 10 animals per condition for three independent experiments. **c**–**d** Behavioral assay showing the total distance moved by fish embryos for at least 12 animals per condition. **e** Average distance (in mm) for each 2-min interval swum by larvae under light-dark period (dark period in gray) at 5 dpf**. f** Cardiac frequency measured in at least 10 animals per condition expressed as beats per minute (BPM) that were calculated by averaging the beats counted four times in 15 s. **g** In situ analysis of liver prox1 and pancreas trypsin markers in zebrafish embryos at 72 hpf, data are reported as average ±SEM. with an unpaired two-tailed Student’s *t* test of four animals per condition. **h** Representative images of developing embryos at 6 hpf and measurements of epiboly area; the yolk was not considered in the analysis. **i** Kinetics of fish growth during the first 7 days of development. The total fish area, excluding the yolk region, was evaluated. **j** Analysis of TRAP1 protein expression profile during the first four days of embryogenesis. Asterisks indicate significant differences (*p < 0.05, **p < 0.01, ***p < 0.001).

We generated Zebrafish TRAP1 knock-out animals using the CRISPR/Cas9 system with a sgRNA targeting TRAP1 exon 6 (Supplementary Fig. 1a–e). Backcrossing of F_0_ founders with wild-type Zebrafish and subsequent screening of offspring larvae by HMA allowed the identification of F_1_ germline carriers (Supplementary Fig. 1b) with a 13 nucleotide deletion (Δ13) in exon 6 (Supplementary Fig. 1c). Homozygous TRAP1 Δ13 fish (Trap1^−/−^) obtained at F_4_ generation (Fig. 1a and Supplementary Fig. 1d, e) were used for experimental purposes.

We found that TRAP1 ablation in Zebrafish is compatible with life as fish reach adulthood and are fertile. The absence of TRAP1 does not affect the structure and fiber organization of muscle, as no differences were detected in muscle birefringence (Fig. 1b and Supplementary Fig. 1f), a physical property of light that indicates the integrity of muscle sarcomeres^25^. In addition, behavioral assays showed that genetic ablation of TRAP1 does not affect locomotor activity (Fig. 1c–d) as well as the normal response to light stimuli of larvae (Fig. 1e). Further, no changes in cardiac functions were detected in TRAP1 knock-out fish (Fig. 1f). Next, we evaluated whether TRAP1 absence affects organogenesis by assessing the development of liver and pancreas at 72 hpf. We found no alteration in liver development at 72 hpf as the expression of Prox1, a specific liver marker, is not altered between TRAP1 knock-out and WT embryos (Fig. 1g). Instead, we observed a delay in pancreas organogenesis, as it is smaller and trypsin expression is reduced in TRAP1 knock-out embryos with respect to their wild-type counterparts (Fig. 1g). We also found a delay in early development of TRAP1 fish. Epiboly, i.e., the spreading of cells out of the blastula to form initial sheets of tissues, was markedly retarded at 6 hpf in TRAP1 knock out larvae (Fig. 1h). A growth delay of TRAP1 knock-out fish was evident at 24 hpf and maintained during the successive developmental stages until 5 dpf (Fig. 1i), and the number of pigments was lower in TRAP1 knock-out fish until 96 hpf (Supplementary Fig. 1g). These differences were gradually reduced at 5 dpf and completely lost at 6–7 dpf (Fig. 1i, and Supplementary Fig. 1g) and adult fish was not smaller when TRAP1 was absent (Supplementary Fig. 1h). These observations are in accord with the changes observed in TRAP1 expression profile during Zebrafish development, as TRAP1 protein declined at 4–5 dpf (Fig. 1j), in accord with RNA-sequencing data collected in Expression Atlas database (http://www.ebi.ac.uk/gxa/experiments/E-ERAD-475) that report a reduction in TRAP1 expression during the first 18 developmental stages, which encompass 5 dpf (Supplementary Fig. 1i).

TRAP1 activity could be critical during Zebrafish development in handling oxidative stress linked to the high rate of proliferation coupled to poorly efficient OXPHOS, in accord with the previously reported antioxidant activity of TRAP1 in cancer cell models^35,36^. Indeed, we found that TRAP1 knock-out fish displayed a higher level of mitochondrial reactive oxygen species (ROS) than their wild-type counterpart (Supplementary Fig. 1j). However, knocking-out TRAP1 is not lethal in Zebrafish and does not hamper its full development, as already observed in mice^37^.

### TRAP1 regulates mitochondrial bioenergetics during Zebrafish embryogenesis

**Fig. 2:**
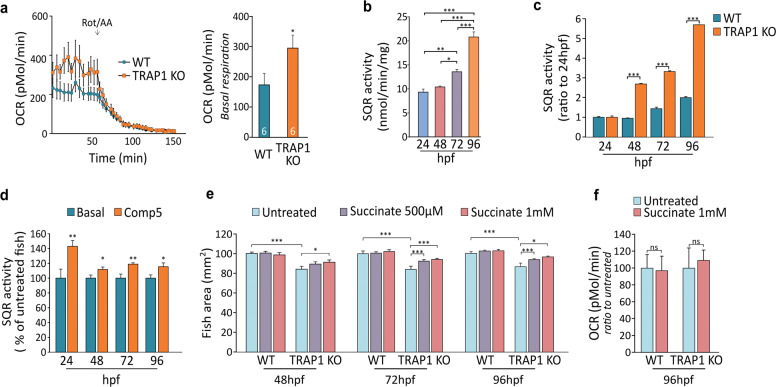
TRAP1 knock-out in Zebrafish affects early embryogenesis stages and mitochondrial bioenergetics. **a** Measurement of oxygen consumption rate (OCR) in TRAP1 wild-type and knock-out embryos at 96 hpf. Respiratory complex I and III inhibitors (2 μM rotenone and 5 μM antimycin A, respectively) were added where indicated. Left: representative traces; right: quantification of basal respiration. **b**–**d** Succinate-CoQ reductase (SQR) enzymatic activity of succinate dehydrogenase (SDH) in total wild-type or TRAP1 knock-out embryo lysates. Where indicated, embryos were treated with the TRAP1 inhibitor compound 5 (100 µM) for 2 h. **e** Kinetics of fish growth from 48 hpf to 96 hpf; where indicated, fish were treated with succinate (500 µM or 1 mM). Succinate was added directly in fish water every day from 24 hpf to 96 hpf. The total fish area, excluding the yolk region, was considered. **f** Quantification of basal mitochondrial respiration in wild-type and TRAP1 knock-out embryos at 96 hpf; where indicated, fish were treated with succinate 1 mM every day from 24 hpf until 96 hpf. Inhibitors of respiratory complex I and III (2 μM rotenone and 5 μM antimycin A, respectively) were added where indicated. Data were normalized to untreated fish and reported as average ±SEM of at least six animals (**a, f**) or 15 animals (**e**) and 60 embryos (in **b–d**) per condition. In **a**, **c**, **d**, **e**, **f** data are reported as average ±SEM with an unpaired two-tailed Student’s *t* test; in (**b**) data are reported as average ±SEM with one-way ANOVA and Bonferroni’s correction of at least four independent experiments; asterisks indicate significant differences (*p < 0.05, ∗∗*p* < 0.01, ***p < 0.001).

TRAP1 displays an inhibitory function on OXPHOS in several tumor cell types^16,36,38^, where it downregulates SDH activity^17^. Therefore, we wondered whether its absence could affect mitochondrial bioenergetics during early development stages of Zebrafish larvae, when OXPHOS activity and oxygen tension are maintained low^20^. We observed that abrogating TRAP1 expression enhanced OCR in whole living fish embryos (Fig. 2a). SDH activity displayed a progressive increase within the first 4 days of development, in keeping with the decline in TRAP1 protein levels recorded in the same period (Fig. 2b). The absence of TRAP1 of pharmacological treatment with the highly specific TRAP1 allosteric inhibitor compound 5 ^39^ increased SDH activity at each developmental stage (Fig. 2c, d), strongly connecting TRAP1 activity with SDH inhibition. Treatment with succinate rescued the developmental delay of TRAP1 knock-out embryos (Fig. 2e), indicating that the TRAP1-dependent accumulation of succinate is essential for the Zebrafish developmental program. Succinate administration did not affect mitochondrial respiration (Fig. 2f), further confirming that it acts downstream to the bioenergetic machinery to impinge upon fish growth.

### TRAP1 is induced in hypoxic conditions

**Fig. 3:**
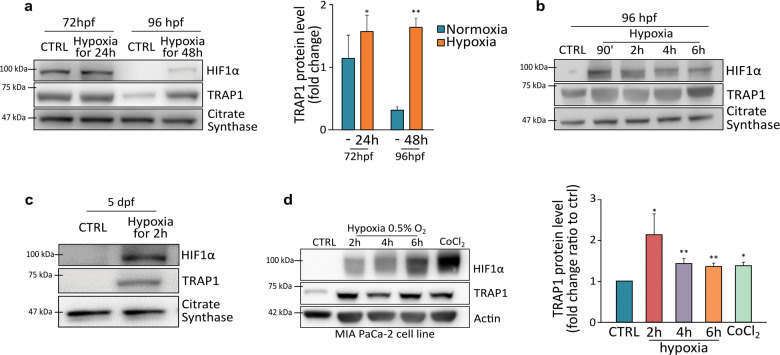
TRAP1 is induced following hypoxia. **a** Western blot analysis (left) and protein quantification (right) of TRAP1 expression level in embryos exposed to hypoxia (5% O_2_) from the stage of 48 hpf for 24 and 48 h. **b** TRAP1 expression profile was analyzed at 96 hpf following short-term hypoxia treatment as indicated. **c** Western blot analysis of TRAP1 expression in Zebrafish at 5 dpf following short-term hypoxia treatment. **d** Western blot analysis and quantification of TRAP1 expression in MIA PaCa-2 human pancreatic adenocarcinoma cells following 2–6 h of hypoxia (0.5% O_2_) or treatment with CoCl_2_ (0.5 mM for 6 h). The mitochondrial protein citrate synthase and actin were used as loading controls for fish and human cells, respectively. Data are reported as average ±SEM of at least three independent experiments with an unpaired two-tailed Student’s *t* test; asterisks indicate significant differences (*p < 0.05, **p < 0.01, ***p < 0.001).

Our data indicate that TRAP1 levels are high during the first days post-fertilization in Zebrafish embryos, when oxygen tension is relatively low. By using a Zebrafish GFP reporter line^22^, we measured a high transcriptional activity of HIF1α in the early stages of embryonic development, which strongly decreased at 4–5 dpf (Supplementary Fig. 2a). These observations suggested the possibility that hypoxia could increase TRAP1 expression. Hence, we exposed 48 hpf Zebrafish larvae to hypoxic conditions for 24 and 48 h, finding an increase in TRAP1 protein levels that was paralleled by the expected raise in HIF1α expression (Fig. 3a). Both TRAP1 and HIF1α were already induced after 90/120 min of hypoxia exposure in fish embryos at 72 hpf (Supplementary Fig. 3a), and at both 96 hpf (Figs. 2b) and 5 dpf (Fig. 2c), where basal TRAP1 protein levels were almost undetectable. The absence of TRAP1 per se is not sufficient to change HIF1α stabilization under normoxia or hypoxia (Supplementary Fig. 3b). We also observed a fast induction of TRAP1 following exposure to hypoxic conditions in several human tumor cell types, encompassing MIA PaCa-2 pancreatic cancer cells (Fig. 3d), U87 and U251 glioblastoma cells, BxPC3 pancreatic carcinoma cells, and ipNF95.6 plexiform neurofibroma cells (Supplementary Fig. 3c–f).

### TRAP1 is regulated in a HIF1α-dependent manner

**Fig. 4:**
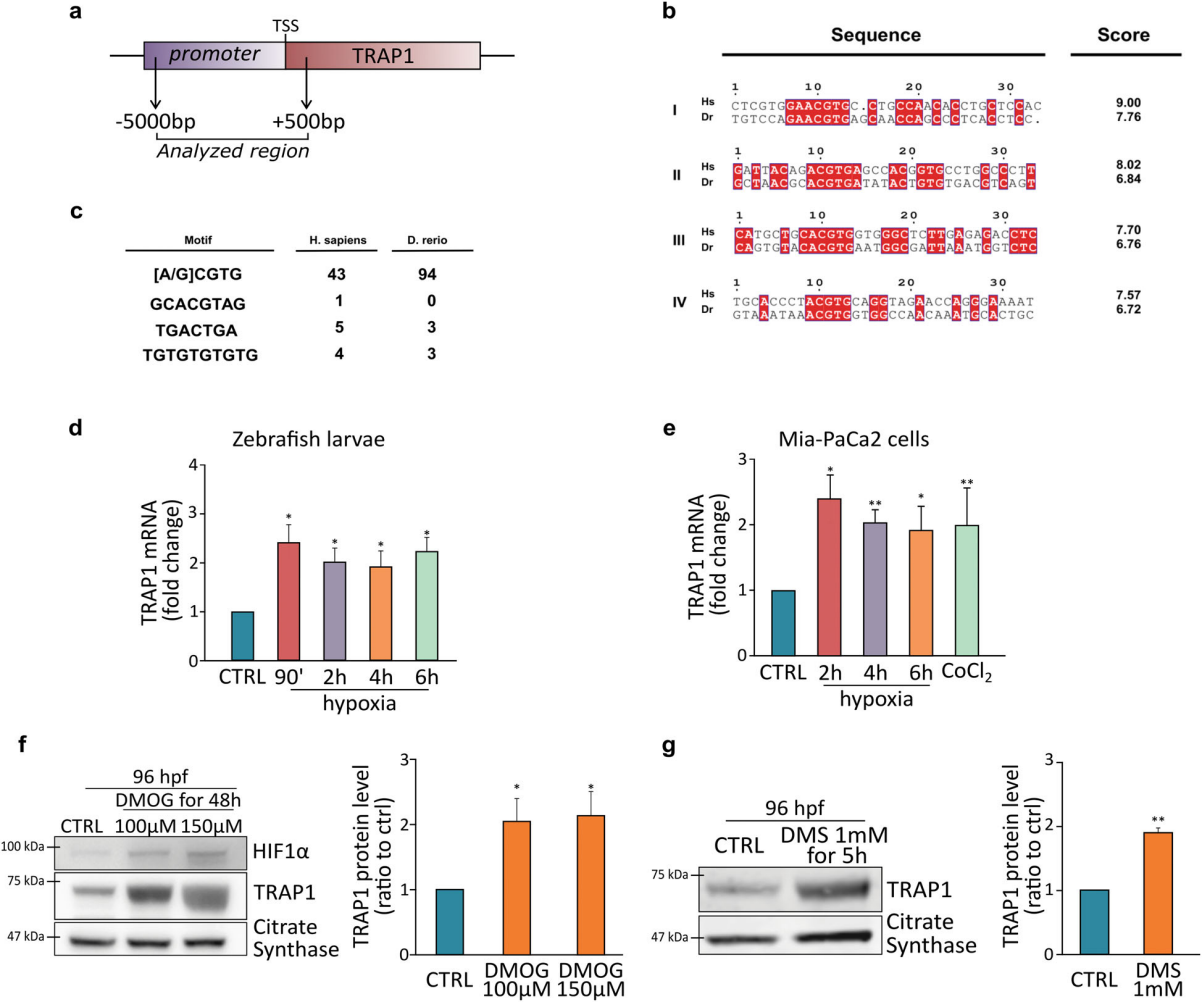
TRAP1 is regulated in a HIF1α-dependent manner. **a** Schematic representation of the TRAP1 promoter region analyzed that encompasses position −5000 bp to +500 bp from the transcription initiation site. **b** List of the four (I–IV) HRE regions more conserved between human (*Homo sapiens*, Hs) and Zebrafish (*Danio rerio*, Dr) TRAP1 promoters. Scoring was calculated using the position-specific matrix. **c** Alternative hypoxia-dependent regulative motifs found within the TRAP1 promoter region of *Homo sapiens* and *Danio rerio*. **d**, **e** Analysis of TRAP1 mRNA expression in wild-type Zebrafish embryos (**d**) and MIA PaCa-2 human pancreatic adenocarcinoma cells (**e**) exposed to hypoxia for the indicated times. Values were normalized for expression of rpl13 (**d**) and actin (**e**) used as housekeeping genes. **f** Western blot analysis and protein quantification of TRAP1 expression in embryos at 96 hpf following a 48 h treatment with 100 and 150 µM dimethyloxalyglycine (DMOG). **g** Western blot analysis and protein quantification of TRAP1 expression in embryos at 96 hpf following a 5 h treatment with 1 mM dimethylsuccinate (DMS). Data are reported as average ±SEM of at least three independent experiments with an unpaired two-tailed Student’s *t* test; asterisks indicate significant differences (*p < 0.05, **p < 0.01, ***p < 0.001).

To evaluate whether TRAP1 induction under hypoxia is caused by its transcriptional upregulation by HIF1α, we searched for hypoxia-responsive elements (HREs), the conserved [A/G]CGTG DNA motifs recognized by HIF1 ^40^, within the TRAP1 gene promoter. As the precise extension of this promoter is still undetermined in Zebrafish, we explored a wide genomic region upstream to the Zebrafish TRAP1 locus (*Danio rerio*, Chromosome 3: 9,602,709-9,659,449), including position −5000 bp and +500 bp from the transcription initiation site of the TRAP1 locus (*Danio rerio*, Chromosome 3:9,597,709-9,603,209) (Fig. 4a). To reduce the probability of identifying misleading motifs, we extended the analysis to the HRE flanking regions assessing a final segment of 33 bp including positions −8 bp up to +20 bp from the canonical HRE^41^. A preliminary screening identified 94 positive hits homogeneously distributed and showing no preference of localization (Supplementary Table 2). In order to determine whether any putative HRE may represent an actual HIF1α binding site, we assigned to each HRE a probability value generated using a position-specific frequency matrix^41^, thus obtaining a final list of 39 highly confident HREs (Supplementary Table 3). A similar search was repeated on the human TRAP1 promoter region (*Homo sapiens*, Chromosome 16: 3,716,198-3,718,509) finding 43 putative HREs, with 31 sequences presenting a significant probability score (Supplementary Table 4). Conservation between *Homo sapiens* and *Zebrafish* was then evaluated by sequence alignment that identified four shared sequences containing the motif [A/G]CGTG (Fig. 4b). These findings are in accord with a HIF1α-dependent regulation of the TRAP1 locus that is evolutionary conserved between Zebrafish and mammals. To reinforce this finding, we extended the analysis by including three supplementary motifs considered to be conserved among hypoxia-responsive genes^42^, finding multiple occurrences of these supplementary hypoxia-related motifs in both human and Zebrafish TRAP1 loci (Fig. 4c). HREs are the minimal *cis*-regulatory elements required for HIF1α *trans*-activation^40^, but hypoxia-dependent gene transcription frequently requires additional transcription factors synergistically acting with HIF1α^43^. GeneCards database (https://www.genecards.org) reported over 30 different transcription factors binding the TRAP1 promoter region. Inspection with STRING database (https://string-db.org/) showed that five of them, i.e., SP1, HDAC1, SIN3A, MAX, MYC, are experimentally validated interactors of HIF1α (Supplementary Fig. 4a). These findings suggest that HIF1α could mediate TRAP1 transcription acting as a co-transcriptional regulator in tandem with additional transcription factors (Supplementary Fig. 4b).

In order to confirm these in silico predictions, we exposed to hypoxia both Zebrafish embryos and human pancreatic adenocarcinoma MIA PaCa-2 cells, finding an increase of TRAP1 mRNA levels (Fig. 4d, e). To further assess whether HIF1α stabilization can prompt TRAP1 induction, we inhibited prolyl hydroxylases (PHDs) by using either dimethyloxalilglycine (DMOG) or dimethyl-succinate (DMS), thus preventing HIF1α priming for proteasomal degradation and eliciting pseudo-hypoxic conditions. Treatment of 48 hpf fish embryos with DMOG or DMS strongly induced TRAP1 protein levels (Fig. 4f, g); moreover, knocking-down HIF1α expression almost ablated TRAP1 induction following hypoxia treatment (Supplementary Fig. 4c).

**Fig. 5:**
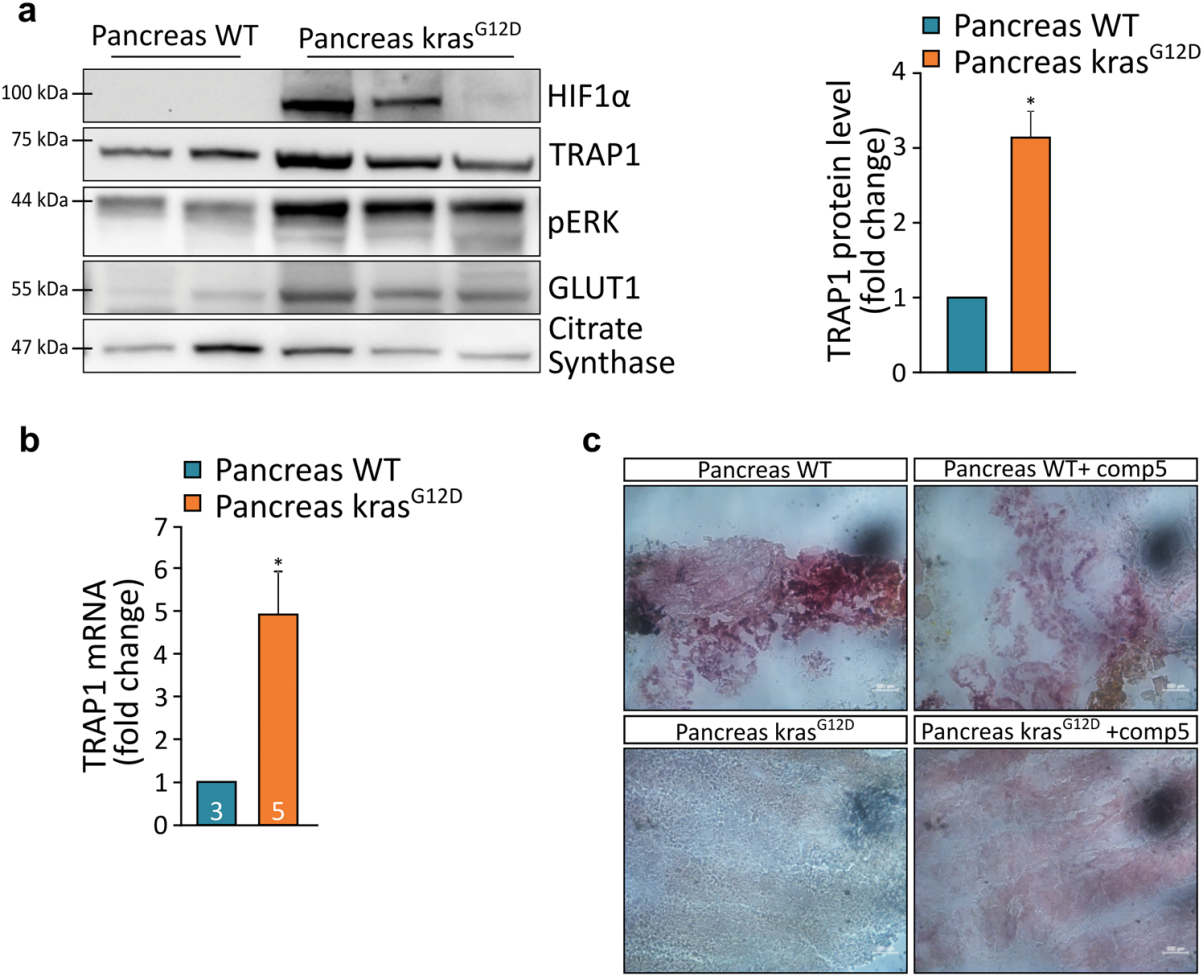
TRAP1 is overexpressed in pancreatic tumors and downregulates SDH activity. **a** Western blot analysis and protein quantification of TRAP1 in wild-type pancreas and in pancreatic tumors driven by KRAS^G12D^ expression (1 year post-fertilization, ypf). Values were normalized for citrate synthase. **b** Analysis of TRAP1 mRNA expression in wild-type pancreas and in pancreatic tumors at 1 ypf. Values were normalized for expression of rpl13 and actin, used as housekeeping genes. The number of animals used in each condition is reported inside column bars. **c** Colorimetric assay (pink staining) of SDH activity in tissue slides of wild-type pancreas and pancreatic tumors with/without the TRAP1 specific inhibitor compound 5 (100 μM, 30 min pre-incubation). In (**a**, **b**), data are reported as average ±SEM of at least four different animals per condition with an unpaired two-tailed Student’s *t* test; asterisks indicate significant differences (*p < 0.05).

Previous reports indicate that HIF1α is stabilized in pancreatic cancer^44–46^. We therefore investigated TRAP1 expression in a Zebrafish model of pancreatic adenocarcinoma induced by expressing in the pancreas eGFP-K-Ras^G12D^ with the conditional Gal4/UAS system under the control of the ptf1a promoter^23^ (Supplementary Fig. 5a). Histological analysis of these fish confirmed the presence of pancreatic adenocarcinoma with mixed acinar and ductal features (Supplementary Fig. 5b). In line with published data, we could confirm an activation of HIF1α in pancreatic tumors, as shown by the nuclear accumulation of the HIF1α reporter protein mcherry fused with the nuclear localization signal (nls; Supplementary Fig. 5a, lower panel), and an enhancement of HIF1α protein levels in cancer samples with respect to wild-type pancreas (Fig. 5a and Supplementary Fig. 5d). TRAP1 expression, which was very low in fish pancreas under physiological conditions (Supplementary Fig. 5c), was strongly increased both at protein and mRNA levels in K-Ras^G12D^-driven pancreatic adenocarcinomas (Fig. 5a, b and Supplementary Fig. 5d). Histochemical investigations of SDH activity on tissue slices showed that SDH activity was strongly reduced in tumor masses compared to the normal pancreas, and incubation with the specific TRAP1 inhibitor compound 5 restored the SDH enzymatic activity in samples of pancreatic tumors (Fig. 5c).

Taken together, our data indicate that TRAP1 is induced in a HIF1α-dependent way under both hypoxic and pseudo-hypoxic conditions, and its induction has important consequences on the in vivo metabolic rewiring of neoplasms.

### TRAP1 regulates mitochondrial respiration during hypoxia

**Fig. 6:**
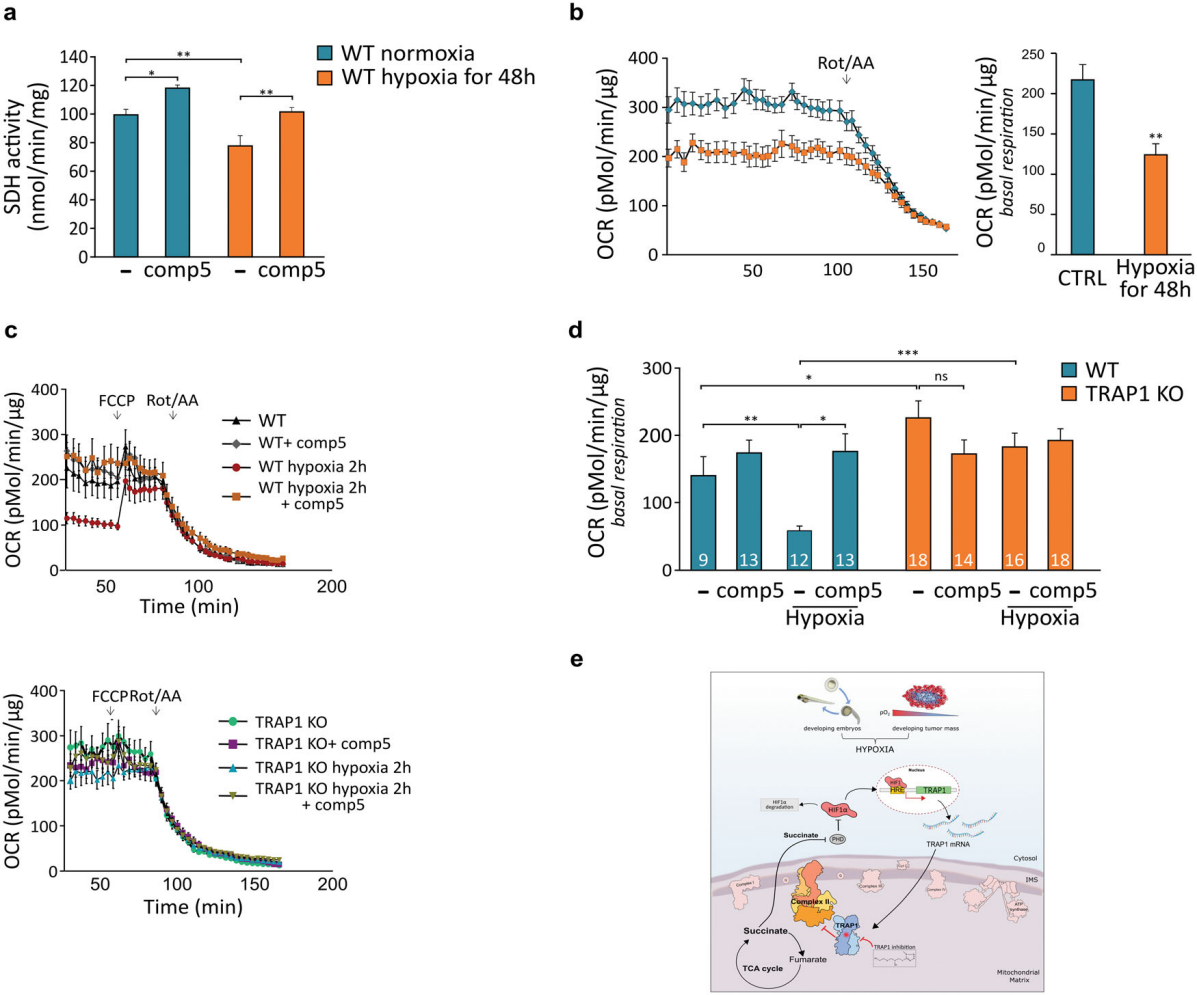
TRAP1 inhibits respiration under hypoxia. **a** Spectrophotometric measurements of SDH activity on wild-type Zebrafish embryos kept in normoxia or hypoxia (5% O_2_) for 48 h. Where indicated, embryos were treated with the specific TRAP1 inhibitor compound 5 (100 μM, 2 h). **b** Assessment of oxygen consumption rate (OCR) in wild-type embryos at 96 hpf, either untreated (blue line and bar) or exposed to hypoxia for 48 h (orange line and bar). Respiratory complex I and III inhibitors (2 μM rotenone and 5 μM antimycin A, respectively) were added where indicated. **c**, **d** OCR measurements in TRAP1 wild-type and knock-out living Zebrafish embryos, either kept in normoxic condition or after 2 h of exposure to hypoxia (5% O_2_). Subsequent additions of the proton uncoupler carbonyl cyanide-4-(trifluoromethoxy)phenylhydrazone (FCCP, 0.5 μM) and of the respiratory complex I and III inhibitors (2 μM rotenone and 5 μM antimycin A, respectively) were carried out as indicated. The specific TRAP1 inhibitor compound 5 (100 μM) was added in fish water 4 h prior to OCR analysis. In fish exposed to hypoxia, the drug was added 2 h before hypoxic treatment and maintained throughout hypoxia exposure. The number of animals used for each condition is reported inside column bars. **e** Schematic representation of the feed-forward crosstalk between HIF1α and TRAP1. In (**a–d**), data are reported as average ±SEM with one-way ANOVA and Bonferroni’s correction of four independent experiments (**a**, **c**, **d**), or at least 20 animals per condition (**b**) with an unpaired two-tailed Student’s *t* test. Asterisks indicate significant differences (*p < 0.05, ∗∗*p* < 0.01, ***p < 0.001).

We then asked whether TRAP1 could contribute to the bioenergetic features of hypoxic cells. Hypoxia slowed down embryo development, an effect exacerbated by the absence of TRAP1 (Supplementary Fig. 6a). As expected, a prolonged exposure (48 h) of Zebrafish embryos to hypoxia lowered both their SDH activity (Fig. 6a) and OCR (Fig. 6b). Treatment with the selective TRAP1 allosteric inhibitor compound 5 (Fig. 6a) or with the non-specific HSP90 family inhibitor 17AAG (Supplementary Fig. 6b) rescued SDH activity to levels comparable to normoxic fish, indicating that SDH inhibition in hypoxic Zebrafish larvae was TRAP1-dependent and reversible. Similarly, the reduction in basal OCR observed by placing embryos under hypoxic conditions was completely lost when we abrogated TRAP1 activity, either by compound 5 administration or in TRAP1 knock-out fish (Fig. 6c upper panel and Fig. 6d). Remarkably, hypoxia could not decrease basal OCR in TRAP1 knock-out embryos, nor the TRAP1-targeting compound 5 affected mitochondrial respiration in normoxic or hypoxic conditions (Fig. 6c lower panel and Fig. 6d). Of note, for technical reasons OCR measurements were performed briefly after returning larvae to normoxia, indicating that hypoxia-mediated respiratory inhibition was maintained following the shift to atmospheric oxygen tension, yet it did not occur in the absence of TRAP1 activity. These observations point towards a primary role of TRAP1 in regulating mitochondrial metabolism of Zebrafish embryos exposed to hypoxic conditions.

## Discussion

In the present study, we demonstrate that the mitochondrial chaperone TRAP1 is a transcriptional target of HIF1α and that TRAP1 is induced in hypoxic conditions both in fish embryos and in tumor models. Under hypoxia, TRAP1 plays a major role in inhibiting respiration.

We have previously found that TRAP1 can prompt the HIF1α transcriptional program via SDH inhibition and the consequent raise in intracellular succinate that inhibits proteasomal degradation of HIF1α^17^. These observations, together with those reported herein, indicate the presence of a feed-forward loop where TRAP1 induces HIF1α, which in turn increases TRAP1 levels (Fig. 6e). This crosstalk could play important roles in coupling the metabolic status of the cell with environmental cues, such as fluctuating oxygen tension. These unstable conditions are experienced by cells not only in pathological conditions, exemplified by the growth of the neoplastic mass in an irregularly vascularized milieu^6,47^, but also in specific physiological settings. During embryonic development, conditions of variable oxygen usage frequently occur when cells must sustain high rates of biomass production during morphogenetic events. Indeed, developing embryos display high levels of aerobic glycolysis^1,3^, highly similar to the Warburg effect that characterizes many cases of tumor growth, and HIF1α plays a master regulatory role in the correct differentiation of organs and tissues^48^.

TRAP1 is a critical regulator of mitochondrial metabolism under stress conditions^36,49^. Here we find that TRAP1 is highly expressed at the beginning of fish embryogenesis, when it exerts an important bioenergetic role. As previously reported in tumor models^16,17,35,39,50,51^, TRAP1 inhibits SDH activity during early Zebrafish development, crucially contributing to a bioenergetic phenotype characterized by low levels of OXPHOS. The absence of TRAP1 causes a delay in early development; this defect is gradually lost during the passage from embryo to larva stage, when oxygen tension increases and TRAP1 levels progressively decline. Notably, succinate treatment rescues developmental defects observed in TRAP1 knock-out fish, pointing towards an essential role of TRAP1 during Zebrafish development via succinate accumulation. These data indicate that TRAP1 is a component of the machinery that connects the efficient utilization of metabolism for developing purposes, albeit the lack of an overt phenotype caused by the homozygous deletion of TRAP1 in Zebrafish suggests that compensatory mechanisms must exist, in accord with what was already observed in TRAP1 knock out mice^52^.

TRAP1 expression increases in both fish embryos and human cancer cells exposed to low oxygen tension. The TRAP1 promoter harbors HIF1α binding sites called HRE in both Zebrafish and human genes, as well as binding sites for five additional transcription factors (SP1, HDAC1, SIN3A, MAX, and MYC), which could cooperate with HIF1α in tuning TRAP1 expression. Interestingly, both SIN3A and MAX regulate the activity of MYC, and scattered evidences suggest that TRAP1 expression is elicited by MYC activity^53,54^. Myc can cooperate with HIF1α to ensure the rapid adaptation to an environment endowed with oxygen paucity by modulating the expression of several target genes involved in metabolic reprogramming, cell cycle, and proliferation^55^. Among HIF1α target genes, heat shock proteins (HSPs) are key during development^56,57^, as they ensure correct protein folding and activity^58,59^ especially under stressful conditions such as hypoxia. The expression profile of the HSP90-family paralog TRAP1 well correlates with that of other HSPs modulated by HIF1α during Zebrafish embryogenesis^60^.

Of note, TRAP1 induction occurs very rapidly also following pseudo-hypoxic stabilization of HIF1α. In cancer, this could be an adaptive mechanism to equip cells in advance for upcoming harsh conditions of nutrient and oxygen shortages. Blocking TRAP1 activity pharmacologically or by knocking-out its expression fully abrogates the inhibition of respiration that characterizes conditions of lower oxygen availability. Instead, in the presence of TRAP1, respiration is inhibited under hypoxia, and this downregulation is maintained even shortly after normal oxygen tension is restored. This finding position TRAP1 as one of the primary inhibitors of mitochondrial respiration under hypoxia. Its bioenergetic consequences could include protection of mitochondria from ROS produced during post-ischemic reperfusion and shaping metabolic responses that rely upon succinate accumulation.

The use of selective inhibitors of TRAP1 chaperone activity^39,61^ has proven instrumental here to demonstrate that these bioenergetic adaptations are rapidly reversible, which probably confers high flexibility in harmonizing respiratory regulation with environmental changes. The chaperone activity of TRAP1 is enhanced by its ERK-mediated phosphorylation^16^, and dysregulated activation of Ras/ERK signaling is mandatory for the malignant growth of a variety of cancer types, including highly aggressive pancreatic adenocarcinoma and malignant peripheral nerve sheath tumors, where we show that TRAP1 has a dramatic inhibitory effect on SDH activity (this study and ref. ^39^). Hence, the possibility of targeting TRAP1 with great precision unlocks the doorway to the development of therapeutic approaches in neoplastic conditions where the metabolic rewiring that it orchestrates is critical for malignant cell viability.

## Supplementary information

Supplemental Figure 1

Supplemental Figure 2

Supplemental Figure 3

Supplemental Figure 4

Supplemental Figure 5

Supplemental Figure 6

Supplementary Table 1 Laquatra et al

Supplementary Table 2 Laquatra et al

Supplementary Table 3 Laquatra et al

Supplementary Table 4 Laquatra et al

## References

[1] Lange C (2016). Relief of hypoxia by angiogenesis promotes neural stem cell differentiation by targeting glycolysis. EMBO J..

[2] Miyazawa H, Aulehla A (2018). Revisiting the role of metabolism during development. Development.

[3] Dunwoodie SL (2009). The role of hypoxia in development of the Mammalian embryo. Dev. Cell.

[4] Pavlova NN, Thompson CB (2016). The emerging hallmarks of cancer metabolism. Cell Metab..

[5] Warburg O (1956). On the origin of cancer cells. Science.

[6] Vander Heiden MG, DeBerardinis RJ (2017). Understanding the intersections between metabolism and cancer biology. Cell.

[7] Agathocleous M (2012). Metabolic differentiation in the embryonic retina. Nat. Cell Biol..

[8] Miyazawa H (2017). Rewiring of embryonic glucose metabolism via suppression of PFK-1 and aldolase during mouse chorioallantoic branching. Development.

[9] Lima A, Burgstaller J, Sanchez-Nieto JM, Rodriguez TA (2018). The mitochondria and the regulation of cell fitness during early mammalian development. Curr. Top. Dev. Biol..

[10] Gordan JD, Thompson CB, Simon MC (2007). HIF and c-Myc: sibling rivals for control of cancer cell metabolism and proliferation. Cancer Cell.

[11] Zhou W (2012). HIF1alpha induced switch from bivalent to exclusively glycolytic metabolism during ESC-to-EpiSC/hESC transition. EMBO J..

[12] Semenza GL (2013). HIF-1 mediates metabolic responses to intratumoral hypoxia and oncogenic mutations. J. Clin. Invest..

[13] Compernolle V (2003). Cardia bifida, defective heart development and abnormal neural crest migration in embryos lacking hypoxia-inducible factor-1alpha. Cardiovasc. Res..

[14] Iyer NV (1998). Cellular and developmental control of O2 homeostasis by hypoxia-inducible factor 1 alpha. Genes Dev..

[15] Kotch LE, Iyer NV, Laughner E, Semenza GL (1999). Defective vascularization of HIF-1alpha-null embryos is not associated with VEGF deficiency but with mesenchymal cell death. Dev. Biol..

[16] Masgras I (2017). Absence of neurofibromin induces an oncogenic metabolic switch via mitochondrial ERK-mediated phosphorylation of the chaperone TRAP1. Cell Rep..

[17] Sciacovelli M (2013). The mitochondrial chaperone TRAP1 promotes neoplastic growth by inhibiting succinate dehydrogenase. Cell Metab..

[18] Kari G, Rodeck U, Dicker AP (2007). Zebrafish: an emerging model system for human disease and drug discovery. Clin. Pharmacol. Ther..

[19] Santoro MM (2014). Zebrafish as a model to explore cell metabolism. Trends Endocrinol. Metab..

[20] Stackley KD, Beeson CC, Rahn JJ, Chan SS (2011). Bioenergetic profiling of zebrafish embryonic development. PLoS ONE.

[21] Kimmel CB, Ballard WW, Kimmel SR, Ullmann B, Schilling TF (1995). Stages of embryonic development of the zebrafish. Dev. Dyn..

[22] Vettori A (2017). Glucocorticoids promote Von Hippel Lindau degradation and Hif-1alpha stabilization. Proc. Natl Acad. Sci. USA.

[23] Schiavone M (2014). Zebrafish reporter lines reveal in vivo signaling pathway activities involved in pancreatic cancer. Dis. Model. Mech..

[24] Gagnon JA (2014). Efficient mutagenesis by Cas9 protein-mediated oligonucleotide insertion and large-scale assessment of single-guide RNAs. PLoS ONE.

[25] Smith LL, Beggs AH, Gupta VA (2013). Analysis of skeletal muscle defects in larval zebrafish by birefringence and touch-evoke escape response assays. J. Vis. Exp..

[26] Thisse C, Thisse B, Schilling TF, Postlethwait JH (1993). Structure of the zebrafish snail1 gene and its expression in wild-type, spadetail and no tail mutant embryos. Development.

[27] Biemar F (2001). Pancreas development in zebrafish: early dispersed appearance of endocrine hormone expressing cells and their convergence to form the definitive islet. Dev. Biol..

[28] Pistocchi A (2008). Crucial role of zebrafish prox1 in hypothalamic catecholaminergic neurons development. BMC Dev. Biol..

[29] MacPhail RC (2009). Locomotion in larval zebrafish: Influence of time of day, lighting and ethanol. Neurotoxicology.

[30] Aken, B. L. et al. The Ensembl gene annotation system. *Database (Oxford)* (2016). 10.1093/database/baw093 PMC4919035 27337980

[31] Zhang Z, Schwartz S, Wagner L, Miller W (2000). A greedy algorithm for aligning DNA sequences. J. Comput. Biol..

[32] Waterhouse AM, Procter JB, Martin DM, Clamp M, Barton GJ (2009). Jalview Version 2−a multiple sequence alignment editor and analysis workbench. Bioinformatics.

[33] Stelzer G (2011). In-silico human genomics with GeneCards. Hum. Genomics.

[34] Szklarczyk D (2017). The STRING database in 2017: quality-controlled protein-protein association networks, made broadly accessible. Nucleic Acids Res..

[35] Guzzo G, Sciacovelli M, Bernardi P, Rasola A (2014). Inhibition of succinate dehydrogenase by the mitochondrial chaperone TRAP1 has anti-oxidant and anti-apoptotic effects on tumor cells. Oncotarget.

[36] Masgras I, Sanchez-Martin C, Colombo G, Rasola A (2017). The chaperone TRAP1 as a modulator of the mitochondrial adaptations in cancer cells. Front. Oncol..

[37] Trnka J, Elkalaf M, Andel M (2015). Lipophilic triphenylphosphonium cations inhibit mitochondrial electron transport chain and induce mitochondrial proton leak. PLoS ONE.

[38] Yoshida S (2013). Molecular chaperone TRAP1 regulates a metabolic switch between mitochondrial respiration and aerobic glycolysis. Proc. Natl Acad. Sci. USA.

[39] Sanchez-Martin C (2020). Rational design of allosteric and selective inhibitors of the molecular chaperone TRAP1. Cell Rep..

[40] Kaluz S, Kaluzova M, Stanbridge EJ (2008). Regulation of gene expression by hypoxia: integration of the HIF-transduced hypoxic signal at the hypoxia-responsive element. Clin. Chim. Acta.

[41] Pescador N (2005). Identification of a functional hypoxia-responsive element that regulates the expression of the egl nine homologue 3 (egln3/phd3) gene. Biochem. J..

[42] Schodel J (2011). High-resolution genome-wide mapping of HIF-binding sites by ChIP-seq. Blood.

[43] Wang GL, Jiang BH, Rue EA, Semenza GL (1995). Hypoxia-inducible factor 1 is a basic-helix-loop-helix-PAS heterodimer regulated by cellular O2 tension. Proc. Natl Acad. Sci. USA.

[44] Hoffmann AC (2008). High expression of HIF1a is a predictor of clinical outcome in patients with pancreatic ductal adenocarcinomas and correlated to PDGFA, VEGF, and bFGF. Neoplasia.

[45] Schofield CJ, Ratcliffe PJ (2004). Oxygen sensing by HIF hydroxylases. Nat. Rev. Mol. Cell Biol..

[46] Wilkes JG (2018). Pharmacologic ascorbate (P-AscH(-)) suppresses hypoxia-inducible factor-1alpha (HIF-1alpha) in pancreatic adenocarcinoma. Clin. Exp. Metastasis.

[47] Ward PS, Thompson CB (2012). Metabolic reprogramming: a cancer hallmark even Warburg did not anticipate. Cancer Cell.

[48] Ietta F (2006). Dynamic HIF1A regulation during human placental development. Biol. Reprod..

[49] Rasola A, Neckers L, Picard D (2014). Mitochondrial oxidative phosphorylation TRAP(1)ped in tumor cells. Trends Cell Biol..

[50] Kowalik MA (2016). Metabolic reprogramming identifies the most aggressive lesions at early phases of hepatic carcinogenesis. Oncotarget.

[51] Sanchez-Martin C (2021). Honokiol bis-dichloroacetate is a selective allosteric inhibitor of the mitochondrial chaperone TRAP1. Antioxid. Redox Signal..

[52] Lisanti S (2014). Deletion of the mitochondrial chaperone TRAP-1 uncovers global reprogramming of metabolic networks. Cell Rep..

[53] Agarwal E (2019). Myc-mediated transcriptional regulation of the mitochondrial chaperone TRAP1 controls primary and metastatic tumor growth. J. Biol. Chem..

[54] Coller HA (2000). Expression analysis with oligonucleotide microarrays reveals that MYC regulates genes involved in growth, cell cycle, signaling, and adhesion. Proc. Natl Acad. Sci. USA.

[55] Cannino G, Ciscato F, Masgras I, Sanchez-Martin C, Rasola A (2018). Metabolic plasticity of tumor cell mitochondria. Front. Oncol..

[56] Hammerer-Lercher A (2001). Hypoxia induces heat shock protein expression in human coronary artery bypass grafts. Cardiovasc. Res..

[57] Mestril R, Chi SH, Sayen MR, Dillmann WH (1994). Isolation of a novel inducible rat heat-shock protein (HSP70) gene and its expression during ischaemia/hypoxia and heat shock. Biochem. J..

[58] Krone PH, Sass JB, Lele Z (1997). Heat shock protein gene expression during embryonic development of the zebrafish. Cell. Mol. Life Sci..

[59] Pechan PM (1991). Heat shock proteins and cell proliferation. FEBS Lett..

[60] Elicker KS, Hutson LD (2007). Genome-wide analysis and expression profiling of the small heat shock proteins in zebrafish. Gene.

[61] Sanchez-Martin C, Serapian SA, Colombo G, Rasola A (2020). Dynamically shaping chaperones. Allosteric modulators of HSP90 family as regulatory tools of cell metabolism in neoplastic progression. Front. Oncol..

