# Supplementary figures and images for: HIF1α-dependent induction of the mitochondrial chaperone TRAP1 regulates bioenergetic adaptations to hypoxia
Source: Cell Death Dis. 2021 May 1;12(5):434. doi: 10.1038/s41419-021-03716-6 (PMC8088431; doi:10.1038/s41419-021-03716-6)

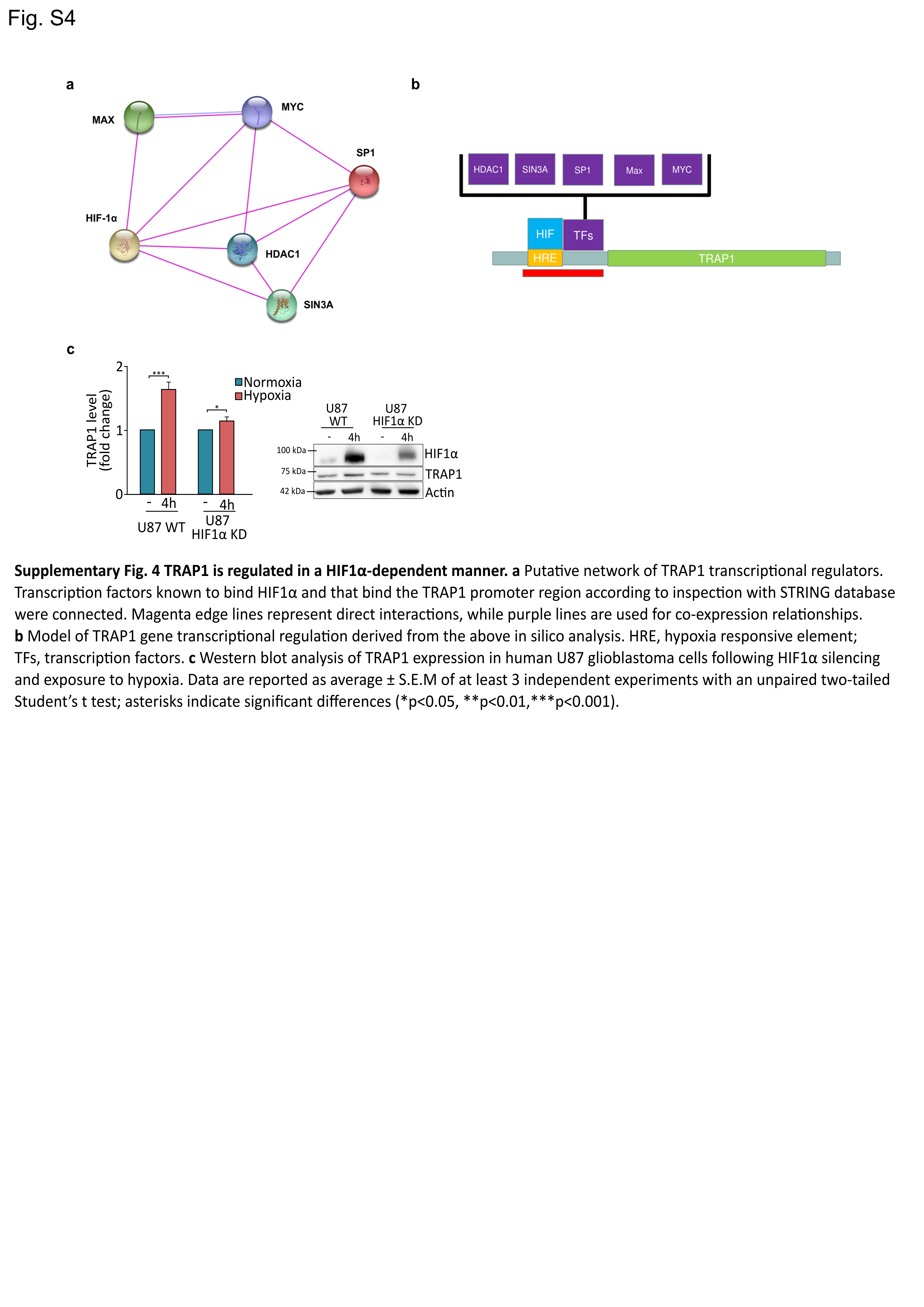

Supplement: Supplementary file 4 — Supplemental Figure 4 [file 41419_2021_3716_MOESM4_ESM.tif]
