# Supplementary material for: HIF1α-dependent induction of the mitochondrial chaperone TRAP1 regulates bioenergetic adaptations to hypoxia
Source: Cell Death Dis. 2021 May 1;12(5):434. doi: 10.1038/s41419-021-03716-6 (PMC8088431; doi:10.1038/s41419-021-03716-6)
Supplement: Supplementary file 7 — Supplementary Table 1 Laquatra et al [file 41419_2021_3716_MOESM7_ESM.docx]

**Supplementary Table 1.** List of primers used to generate and to screen the Zebrafish TRAP1 knock-out fish, and primers used for real-time PCR

| **Generation of Zebrafish TRAP1 KO line** | |
| --- | --- |
| gRNA Exon 6 | CGCTCTGCAGAATCAAGCGGAGG |
| SP6 promoter | ATTTAGGTGACACTATA |
| overlap region | GTTTTAGAGCTAGAAATAGCAAG |
| Fw_TRAP1exon6 | GGGATGAACAAAGAGGATCTGGTTTC |
| Rv_TRAP1exon6 | GGAGGTTTATTCTCAGTCGGCAGAAG |
| **Primers for rtPCR** | |
| Fw_rpl13a | CTCGGTCGTCTTTCCGCTATT |
| Rv_rpl13a | ATACCCTCACACCTCACCAC |
| ZfwTRAP1 | GCACTCGTTCAGAGAGTCACCAA |
| ZrvTRAP1 | ACTGTGATCATGGCCGGGTG |
| Fw_hTRAP1 | GCACTCGTTCAGAGAGTCACCAA |
| Rv_hTRAP1 | ACTGTGATCATGGCCGGGTG |
| Fw_hActin | GAGCGTTTCCGTTGCCCC |
| Rv_hActin | ATACCGCAAGATTCCATACCCAGG |
| Fw_hSDHB | CAGTCGGAGACAAACCTCGCA |
| Rv_hSDHB | CTTGATGAGTGCGTCCAGAACC |
| shHIF1α_10819 | CCGGTGCTCTTTGTGGTTGGATCTACTCGAGTAGATCCAACCACAAAGAGCATTTTT |
| shHIF1α_3809 | CCGGCCAGTTATGATTGTGAAGTTACTCGAGTAACTTCACAATCATAACTGGTTTTT |
