# Supplementary material for: HIF1α-dependent induction of the mitochondrial chaperone TRAP1 regulates bioenergetic adaptations to hypoxia
Source: Cell Death Dis. 2021 May 1;12(5):434. doi: 10.1038/s41419-021-03716-6 (PMC8088431; doi:10.1038/s41419-021-03716-6)
Supplement: Supplementary file 8 — Supplementary Table 2 Laquatra et al [file 41419_2021_3716_MOESM8_ESM.docx]

**Supplementary Table 2.** 94 positive sequences obtained in the preliminary screening of Zebrafish TRAP1 promoter. The sequences are homogeneously distributed, showing no preference of localization.

| **Sequence** | **Score** |
| --- | --- |
| ATAGCAGTGCGTGACTGTCAGCTGACGCGCTCC | 7.89 |
| \| GGGGCAGTACGTGATGGAATAATCGTTTATCTC \|  \| \| --- \| --- \| | 7.88 |
| \| TAATCCACACGTGACTCCAGCTCCACTCTCACA \|  \| \| --- \| --- \| | 7.83 |
| \| TGTCCAGAACGTGAGCAACCAGCCCTCACCTCC \|  \| \| --- \| --- \| | 7.76 |
| \| TGACAGTCACGTGGCGTCGGCGCAGCATCAATC \|  \| \| --- \| --- \| | 7.45 |
| \| TTTGGTGGGCGTGGTGTAAGACAGAAAGTTTCA \|  \| \| --- \| --- \| | 7.22 |
| \| GGCGCCAGACGTGCACTTTGTAGATTTTTAATG \|  \| \| --- \| --- \| | 6.87 |
| \| GCTAACGCACGTGATATACTGTGTGACGTCAGT \|  \| \| --- \| --- \| | 6.84 |
| \| GCTAACGCACGTGATATACTGTGTGACGTCAGT \|  \| \| --- \| --- \| | 6.84 |
| \| CAGTGTACACGTGAATGGCGATTAAATGGTCTC \|  \| \| --- \| --- \| | 6.76 |
| \| GTAAATAAACGTGGTGGCCAACAAATGCACTGC \|  \| \| --- \| --- \| | 6.72 |
| \| CTATACCCACGTGACTGCTTCAAATTTTGATTC \|  \| \| --- \| --- \| | 6.7 |
| \| ACAGATGAACGTGAGCTAACAAAATCAATATGC \|  \| \| --- \| --- \| | 6.69 |
| \| TGCCTCAAACGTGTGTGGTTCTGCGGGCCTGCA \|  \| \| --- \| --- \| | 6.64 |
| \| CAGAGGACACGTGAATACCCACTGTCTAAGTAC \|  \| \| --- \| --- \| | 6.63 |
| \| TAAAGCATACGTGGGAGGAAAGGGGGATAAACT \|  \| \| --- \| --- \| | 6.62 |
| \| CGTTCTAGACGTGCACGCGCTTATTCCGGAAAT \|  \| \| --- \| --- \| | 6.57 |
| \| CAAAATACACGTGTTTGTTGTCATGTGGTGAAC \|  \| \| --- \| --- \| | 6.51 |
| \| CCATCATCACGTGATCAGTGACGTTCGAAGCTT \|  \| \| --- \| --- \| | 6.50 |
| \| TATTGAGGACGTGAAGCTTAAGTCAGGCCCTTT \|  \| \| --- \| --- \| | 6.45 |
| \| TATAACTAACGTGCAAAAGATATAGCCCTTTTC \|  \| \| --- \| --- \| | 6.40 |
| \| TTGGGAGGGCGTGTGTTCTAAAAGGTTGAAAAA \|  \| \| --- \| --- \| | 6.39 |
| \| AGTGCAGTGCGTGCACGTTGTGTTACTGACTCT \|  \| \| --- \| --- \| | 6.38 |
| \| GCATGAGAACGTGCATGAAGACATTTTTACATG \|  \| \| --- \| --- \| | 6.37 |
| \| TGTGTAAAACGTGCTGGATAAGTTGGCGGTTCA \|  \| \| --- \| --- \| | 6.33 |
| \| TGTGTAAAACGTGCTGGATAAGTTGGCGGTTCA \|  \| \| --- \| --- \| | 6.33 |
| \| TAAATAAGACGTGCCTGTTGTTTTGGTCTCTGC \|  \| \| --- \| --- \| | 6.32 |
| \| TTCTTATGGCGTGACCTAAAGAGAGAAGAACTC \|  \| \| --- \| --- \| | 6.27 |
| \| AATGCTGGGCGTGTCATCGTGATTGACAGCTGT \|  \| \| --- \| --- \| | 6.25 |
| \| CATGATATGCGTGTGTGTGCTGGCGCTCACAGG \|  \| \| --- \| --- \| | 6.22 |
| \| TTCAGGTCACGTGACTAAAGTGTTGTGAAACAC \|  \| \| --- \| --- \| | 6.19 |
| \| TGCCGCTTACGTGCTCGCTACATTGTCACAACG \|  \| \| --- \| --- \| | 6.19 |
| \| ATATGATGACGTGTGCAGGGGCTGTAGTGCTGT \|  \| \| --- \| --- \| | 6.13 |
| \| ACCTGGTCACGTGACTAAAGCAATTCAAAGCAT \|  \| \| --- \| --- \| | 6.12 |
| \| AAATAGCAACGTGCCAGCAATACACCTCAACAC \|  \| \| --- \| --- \| | 6.12 |
| \| GGACGCAGGCGTGACGGCACTGATCCTGCGACG \|  \| \| --- \| --- \| | 6.11 |
| \| CTATAGCTACGTGGGAGGTTTCTAAACGACGCT \|  \| \| --- \| --- \| | 6.02 |
| \| GCAACACAACGTGGCGTCTCTGTGGTGTAAACA \|  \| \| --- \| --- \| | 6.02 |
| \| TAATCCACACGTGATCCAGCTGAGCTCTCACAG \|  \| \| --- \| --- \| | 6.00 |
| \| CATACTATACGTGTACTTCACATTAAACCTTTT \|  \| \| --- \| --- \| | 5.97 |
| \| CCAACACAACGTGGCGTCTCTGTTGTCTAAACA \|  \| \| --- \| --- \| | 5.96 |
| \| CTTTCAGAGCGTGTCAGGACGCGTTTTTGCTCA \|  \| \| --- \| --- \| | 5.89 |
| \| GTAATCTAACGTGCTAACCACTGGGCCACAACG \|  \| \| --- \| --- \| | 5.83 |
| \| AAAACAGCACGTGCTCTTTTGCGCTTTTAAAAA \|  \| \| --- \| --- \| | 5.81 |
| \| CGTTGAGTGCGTGAAGTGTCCATCATTACACAC \|  \| \| --- \| --- \| | 5.78 |
| \| CAAAGAAAACGTGTCGTAATGTTCACTATGCAG \|  \| \| --- \| --- \| | 5.76 |
| \| GTTTTCACACGTGTACAACTGGCAAGGGGGCAG \|  \| \| --- \| --- \| | 5.76 |
| \| CATAATGGGCGTGGAGCTCCACGATCAGAGGCA \|  \| \| --- \| --- \| | 5.72 |
| \| AGAAAGCCGCGTGTGTTATTCCGGTCACTAAAT \|  \| \| --- \| --- \| | 5.72 |
| \| TTTTTGTTGCGTGGGTGTTGAGATCCTGATCTT \|  \| \| --- \| --- \| | 5.70 |
| \| AGGAGTGGGCGTGGCCAGCAGAGCAGGGGAGAA \|  \| \| --- \| --- \| | 5.69 |
| \| TTTACCATACGTGTGTCAGTCGGTAATAGTCTG \|  \| \| --- \| --- \| | 5.66 |
| \| TTTACCATACGTGTGTCAGTCGGTAATAGTCTG \|  \| \| --- \| --- \| | 5.66 |
| \| TGAACTCCACGTGACAATTACAGGCAGGTGTGT \|  \| \| --- \| --- \| | 5.63 |
| \| TATCTTCTACGTGCCAGAAATGGTGAGTGAATA \|  \| \| --- \| --- \| | 5.63 |
| \| GAGTATGTGCGTGTCGAGTCGGCAGGTGGACAG \|  \| \| --- \| --- \| | 5.57 |
| \| TTTTGATGACGTGGATTGGGAACAGTTTTGGCT \| \| --- \| | 5.52 |
| \| TGTCTCTTACGTGCTCGCTACATTGTCACAACG \|  \| \| --- \| --- \| | 5.51 |
| \| TTTATACAACGTGCCTAACCTCAGAAAGTGAAA \|  \| \| --- \| --- \| | 5.48 |
| \| GTAATGTGACGTGCTAAAACAAGTGAGCAAGAT \|  \| \| --- \| --- \| | 5.40 |
| \| GTGTTATTACGTGAAGATGCGGGACTTTAGCGC \|  \| \| --- \| --- \| | 5.39 |
| \| AATGTAAGACGTGAGAAGGCTGTAGCAACGTTG \|  \| \| --- \| --- \| | 5.38 |
| \| ACTTTAGTGCGTGTTGGACGTTTGTGTTTGAAC \|  \| \| --- \| --- \| | 5.34 |
| \| CATCTACTGCGTGAGAATGAGAGATAATGGAAC \|  \| \| --- \| --- \| | 5.33 |
| \| CTTTCAAAGCGTGTTGTGTAGGCTTGTAGTCTA \|  \| \| --- \| --- \| | 5.33 |
| \| TTAATTGGACGTGAATTGAAATACAACAACAAA \|  \| \| --- \| --- \| | 5.25 |
| \| TTTGGGGGGCGTGGTTGATTTTACATAAAGCGT \|  \| \| --- \| --- \| | 5.22 |
| \| CAGTGAAAACGTGACTAGGCTGGACAGTCCAGG \|  \| \| --- \| --- \| | 5.20 |
| \| ATACTGTGACGTGGTGGCGCAGTAGGTAGTGCT \|  \| \| --- \| --- \| | 5.01 |
| \| ATACTGTGACGTGGTGGCGCAGTAGGTAGTGCT \|  \| \| --- \| --- \| | 5.01 |
| \| TCTCTTTCACGTGTCTTTTGATTCTCAACTTGT \|  \| \| --- \| --- \| | 5.01 |
| \| TGCTCTAAGCGTGTATTTTTCATGCAATGTTTG \|  \| \| --- \| --- \| | 4.95 |
| \| CTGAAGCCACGTGGAGTTTACTGACTTTTTGGT \|  \| \| --- \| --- \| | 4.91 |
| \| ATTATATCACGTGCTCCTCTCGAAATTAGTTTA \|  \| \| --- \| --- \| | 4.84 |
| \| AACATAATGCGTGCAATACACATTTTAGGTTGA \|  \| \| --- \| --- \| | 4.84 |
| \| AATGTAAGACGTGAAAAGGCTGTAGCAACGTTG \|  \| \| --- \| --- \| | 4.82 |
| \| TTACAACAACGTGATTTTAACGAAGTATGATAA \|  \| \| --- \| --- \| | 4.77 |
| \| AAGAAATAACGTGCTCCGTTTTTCTGCGCTCCA \|  \| \| --- \| --- \| | 4.69 |
| \| TGGTTACAGCGTGATTTATCAATAACTGGTAGA \|  \| \| --- \| --- \| | 4.67 |
| \| CATTAACTGCGTGTCCCATATTGTAAAGTGTGA \|  \| \| --- \| --- \| | 4.67 |
| \| TGAAAAAGACGTGATAGTGTCATTAGAAACTGA \|  \| \| --- \| --- \| | 4.64 |
| \| TCATTTTCACGTGGAACTGAGCTTTGCAGAGGC \|  \| \| --- \| --- \| | 4.62 |
| \| AAAAAGTAACGTGCATATTATAGGAACATGTGC \|  \| \| --- \| --- \| | 4.51 |
| \| AATTGTGTGCGTGCATCTAAATGTAAGTTTCTT \|  \| \| --- \| --- \| | 4.45 |
| \| AATTGTGTGCGTGCATCTAAATGTAAGTTTCTT \|  \| \| --- \| --- \| | 4.45 |
| \| ACTCTTAAACGTGTCTATACATACCAAACATAT \|  \| \| --- \| --- \| | 4.14 |
| \| AATCAAGCGCGTGTTATTATTATCAGCTCAAGA \|  \| \| --- \| --- \| | 4.14 |
| \| AAAAGTGGGCGTGTCCAGCTCTGTTTAGGGGGG \|  \| \| --- \| --- \| | 4.13 |
| \| AAAAGTGGGCGTGTCCAGCTCTGTTTAGGGGGG \|  \| \| --- \| --- \| | 4.13 |
| \| TTTATAATGCGTGTGTGGTCTTGGTATTCATCA \|  \| \| --- \| --- \| | 3.98 |
| \| TTTAAACAGCGTGATTCAAAACGACTCTTGCAT \|  \| \| --- \| --- \| | 3.98 |
| \| TAGTATGTGCGTGTAGTGTATTTTTTAGTAGAT \|  \| \| --- \| --- \| | 3.95 |
| \| GTGATTCAACGTGTACATTTAATAATGTTAAAT \|  \| \| --- \| --- \| | 3.33 |
| \| TTACAGCAGCGTGTCATTCGATTCCTGTTGGAC \|  \| \| --- \| --- \| | 3.26 |
