# Supplementary material for: HIF1α-dependent induction of the mitochondrial chaperone TRAP1 regulates bioenergetic adaptations to hypoxia
Source: Cell Death Dis. 2021 May 1;12(5):434. doi: 10.1038/s41419-021-03716-6 (PMC8088431; doi:10.1038/s41419-021-03716-6)
Supplement: Supplementary file 9 — Supplementary Table 3 Laquatra et al [file 41419_2021_3716_MOESM9_ESM.docx]

**Supplementary Table 3.** List of the top ranking HREs identified within position -5000bp and +500bp from the zebrafish TRAP1 transcription initiation site (Dario rerio, Chromosome 3: 9,602,709-9,659,449) with associated probability score.

| **Sequence** | **Score** |
| --- | --- |
| ATAGCAGTGCGTGACTGTCAGCTGACGCGCTCC | 7.89 |
| GGGGCAGTACGTGATGGAATAATCGTTTATCTC | 7.88 |
| TAATCCACACGTGACTCCAGCTCCACTCTCACA | 7.83 |
| TGTCCAGAACGTGAGCAACCAGCCCTCACCTCC | 7.76 |
| TGACAGTCACGTGGCGTCGGCGCAGCATCAATC | 7.45 |
| TTTGGTGGGCGTGGTGTAAGACAGAAAGTTTCA | 7.22 |
| GGCGCCAGACGTGCACTTTGTAGATTTTTAATG | 6.87 |
| GCTAACGCACGTGATATACTGTGTGACGTCAGT | 6.84 |
| GCTAACGCACGTGATATACTGTGTGACGTCAGT | 6.84 |
| CAGTGTACACGTGAATGGCGATTAAATGGTCTC | 6.76 |
| GTAAATAAACGTGGTGGCCAACAAATGCACTGC | 6.72 |
| CTATACCCACGTGACTGCTTCAAATTTTGATTC | 6.70 |
| ACAGATGAACGTGAGCTAACAAAATCAATATGC | 6.69 |
| TGCCTCAAACGTGTGTGGTTCTGCGGGCCTGCA | 6.64 |
| CAGAGGACACGTGAATACCCACTGTCTAAGTAC | 6.63 |
| TAAAGCATACGTGGGAGGAAAGGGGGATAAACT | 6.62 |
| CGTTCTAGACGTGCACGCGCTTATTCCGGAAAT | 6.57 |
| CAAAATACACGTGTTTGTTGTCATGTGGTGAAC | 6.51 |
| CCATCATCACGTGATCAGTGACGTTCGAAGCTT | 6.50 |
| TATTGAGGACGTGAAGCTTAAGTCAGGCCCTTT | 6.45 |
| TATAACTAACGTGCAAAAGATATAGCCCTTTTC | 6.40 |
| TTGGGAGGGCGTGTGTTCTAAAAGGTTGAAAAA | 6.39 |
| AGTGCAGTGCGTGCACGTTGTGTTACTGACTCT | 6.38 |
| GCATGAGAACGTGCATGAAGACATTTTTACATG | 6.37 |
| TGTGTAAAACGTGCTGGATAAGTTGGCGGTTCA | 6.33 |
| TGTGTAAAACGTGCTGGATAAGTTGGCGGTTCA | 6.33 |
| TAAATAAGACGTGCCTGTTGTTTTGGTCTCTGC | 6.32 |
| TTCTTATGGCGTGACCTAAAGAGAGAAGAACTC | 6.27 |
| AATGCTGGGCGTGTCATCGTGATTGACAGCTGT | 6.25 |
| CATGATATGCGTGTGTGTGCTGGCGCTCACAGG | 6.22 |
| TTCAGGTCACGTGACTAAAGTGTTGTGAAACAC | 6.19 |
| TGCCGCTTACGTGCTCGCTACATTGTCACAACG | 6.19 |
| ATATGATGACGTGTGCAGGGGCTGTAGTGCTGT | 6.13 |
| ACCTGGTCACGTGACTAAAGCAATTCAAAGCAT | 6.12 |
| AAATAGCAACGTGCCAGCAATACACCTCAACAC | 6.12 |
| GGACGCAGGCGTGACGGCACTGATCCTGCGACG | 6.11 |
| CTATAGCTACGTGGGAGGTTTCTAAACGACGCT | 6.02 |
| GCAACACAACGTGGCGTCTCTGTGGTGTAAACA | 6.02 |
| TAATCCACACGTGATCCAGCTGAGCTCTCACAG | 6.00 |
