# Supplementary material for: HIF1α-dependent induction of the mitochondrial chaperone TRAP1 regulates bioenergetic adaptations to hypoxia
Source: Cell Death Dis. 2021 May 1;12(5):434. doi: 10.1038/s41419-021-03716-6 (PMC8088431; doi:10.1038/s41419-021-03716-6)
Supplement: Supplementary file 10 — Supplementary Table 4 Laquatra et al [file 41419_2021_3716_MOESM10_ESM.docx]

**Supplementary Table 4.** List of the top ranking HREs identified within the human TRAP1 promoter region (Homo sapiens, Chromosome 16: 3,716,198-3,718,509) with associated probability score.

| **Sequence** | **Score** |
| --- | --- |
| CTCGTGGAACGTGCCTGCCAACACCTGCTCCAC | 9.00 |
| GAGGCCAGACGTGTCCTCAGTTCAGTGGGTGTC | 8.75 |
| CGGAGCCCACGTGAGAACCAGAGCTGTGGAGGC | 8.25 |
| AACCCAGCACGTGTGCTAAGAGGGAAGCAGGGA | 8.07 |
| GATTACAGACGTGAGCCACGGTGCCTGGCCCTT | 8.02 |
| CCACCTGCACGTGCGCTACCTGACTGGACCGAA | 7.73 |
| CATGCTGCACGTGGTGGGCTCTTGAGAGACCTC | 7.70 |
| TGCACCCTACGTGCAGGTAGAACCAGGGAAAAT | 7.57 |
| CATGTCACACGTGCACACCTACAAAGACCTAGA | 7.52 |
| GATTACAGACGTGAGCCACCATACCTGGCCTCT | 7.52 |
| CAGGCCCGACGTGGTAGCTCATGTCTGTAATCC | 7.45 |
| CTCGTCCCACGTGCTGAGCACACGTAGGGGGTT | 7.21 |
| CGAGCAACACGTGCCGAGCAGGGACCCACAGGG | 7.07 |
| AGCTTAGTACGTGAAGACATGCCTTGGTTCCTA | 6.99 |
| AAGGTGGCACGTGCCTGTAGTCCCAGCTCCTGG | 6.98 |
| CTATCACTACGTGGTCAGTGAGCCACTGGGACG | 6.96 |
| TTAGCCAGACGTGGTGGCGCACTTCTAGTTATC | 6.96 |
| CAGATGCTACGTGCAGGGCTGTCCTTGGAGGGC | 6.95 |
| CCTGGCCAACGTGGTGAAACCCCATCTCTACTA | 6.86 |
| GGTCCTCTACGTGGTGCCAGCTGTTTGGCTTTC | 6.84 |
| CTCACACGACGTGGCTGTCTCCACAGAGGTCAG | 6.83 |
| AGGGCGGCACGTGTTCTGCTTGTGCCCTGGGAG | 6.79 |
| CCCCACACACGTGGTCACCCTCCTCCTGCTGCT | 6.59 |
| GCCACCACACGTGCAAGGGACACGGGATAGGAA | 6.55 |
| GGGTTTTGACGTGCTGGAGGCTGTCCTAGACTT | 6.51 |
| TAATTCCCACGTGTTGTGGGAAGGACCTGGTGG | 6.51 |
| TATCAGCAACGTGAAAATGGACTAATACACTCC | 6.40 |
| GGTGGGCCACGTGGCTGGGCTCCCCACACACCA | 6.38 |
| TCCCAGCTACGTGGGAGGCGAAGTCAGGAGAAT | 6.14 |
| CCCCAGACACGTGGACAAGAATACTCAGAGCAG | 6.07 |
| AATACCGCACGTGAACAAATATTTAAGTACTCA | 6.00 |
| TTTGCAATACGTGATGGTGTCAAGGATATGTGG | 5.90 |
| TGAATTTTACGTGTTAAAAGGTGAATTCTGTGA | 5.85 |
| AAGTGCAAACGTGATTATCAGGTGCAGTTTTTA | 5.69 |
| AAAGAAACACGTGCTAGCAACCCACCTATGCGG | 5.69 |
| AAGACGTAACGTGGGGGTCAAGTCACAGTTAGG | 5.69 |
| TCAGTACCACGTGCTGTGACCCTCCCTTCATGT | 5.59 |
| CTCATGAGACGTGAAGATGAAACTCAAATTTCA | 5.45 |
| CATTCTACACGTGAAGAGCTAAGTCACAGAGAG | 5.44 |
| AGTGAGCAACGTGAGACTTCAGCAAACACCAAG | 5.20 |
| GCATAGTGACGTGCACCTGTAGTCACGCCTCAG | 5.19 |
| CTGAAGTTACGTGTCCATCTGTTAATTATAACT | 5.16 |
| GATGAAACACGTGTTTATGCAGGCTCAGCACAG | 5.15 |
